# Vincristine could partly suppress stromal support to T-ALL blasts during pegylated arginase I treatment

**DOI:** 10.1186/2162-3619-2-11

**Published:** 2013-04-10

**Authors:** Fung Kwong-Lam, Chan Godfrey Chi-Fung

**Affiliations:** 1 Department of Paediatrics & Adolescent Medicine, Li Ka Shing Faculty of Medicine, The University of Hong Kong, Hong Kong, SAR, China

**Keywords:** Arginase, Bone marrow, Lymphoid leukemic cells, Mesenchymal stromal cell, Ornithine transcarbamylase, Stromal suppression

## Abstract

**Background:**

Relapsed T-lineage acute lymphoblastic leukemia (T-ALL) has been an incurable disease. Recent reports showed that an L-arginine depleting enzyme, pegylated arginase (BCT-100) may be effective against T-ALL cells. On the other hand, studies including ours had shown the symbiosis of ALL blasts and human mesenchymal stromal cells (hMSCs) in bone marrow microenvironment during L-asparaginase treatment. As L-asparaginase and BCT-100 both act by depleting lymphoid cells of specific amino acid, we hypothesized that hMSCs may also protect T-ALL blasts from BCT-100 treatment in co-culture and such protection may be abrogated by pre-treating hMSCs with vincristine (VCR).

**Methods:**

XTT assay was used to test sensitivities of T-ALL cell lines and hMSCs to BCT-100. Apoptosis of T-ALL cell lines with or without BCT-100 treatment were tested by annexin V / propidium iodide (AV/PI) assay using flow cytometer. Western blotting was performed to analyze the expression of ornithine transcarbamylase (OTC), an enzyme involved in L-arginine metabolism which may account for BCT-100 resistance.

**Results:**

hMSCs were resistant to BCT-100 while CCRF-CEM, Jurkat and MOLT-4 were very sensitive to it. hMSCs could protect all the three cell lines from BCT-100 treatment in transwell co-culture. All the 3 T-ALL cell lines were also found to be rescued by an L-arginine precursor citrulline, while the breakdown product of BCT-100, ornithine only had limited salvaging effect on CCRF-CEM but not Jurkat and MOLT-4. Both hMSCs and 3 T-ALL cell lines express citrulline synthesis enzyme, ornithine transcarbamylase (OTC) at basal level while only hMSCs could express OTC at relatively higher level under BCT-100 treatment. Treating hMSCs with vincristine before co-culturing with T-ALL could resume the cytotoxicity of BCT-100 to CCRF-CEM and MOLT-4 cells.

**Conclusions:**

Our results suggest a possible strategy to overcome resistance to BCT-100 from cancer microenvironments by suppressing hMSCs either in marrow or in the perivascular niche using vincristine.

## Background

Acute lymphoblastic leukemia (ALL) is the commonest form of pediatric malignancies which originates from lymphoid precursors [1]. ALL can be divided into 2 sub-types, B-lineage ALL (B-ALL) accounts for 85% of all cases and the remaining 15% are T-lineage ALL (T-ALL) [2]. With the advances in anti-cancer drugs development and treatment regimen design, the long term event free survival of childhood ALL can be up to 85% these days [3]. However, T-ALL is known to have less favorable prognosis and relapse is more common probably due to emergence of chemo-resistance [4]. For patients with relapsed T-ALL, there is still no effective curative strategy up to the moment.

L-asparaginase is a commonly used chemotherapeutic agent in both B-ALL and T-ALL. Its main mechanism is by depletion of L-asparagine, a non-essential amino acid. Leukemic blasts lack L-asparaginase resulting in chemoresistance. It has been shown that mesenchymal stromal cells (MSCs) may contribute to L-asparaginase resistance of B-ALL blasts in bone marrow microenvironment by secreting asparagine [5]. On the other hand, MSCs have a peculiar chemo-sensitivity pattern. MSCs are resistant to most of the commonly used chemotherapeutic agents for leukemia, but remain sensitive to anti-microtubule agents such as paclitaxel and vincristine (VCR) [6]. In almost all currently adopted protocols for childhood ALL, it is recommended to administer VCR 1 or 2 days prior to L-asparaginase administration [7]. Traditional view was that such practice can suppress the allergic reaction to asparaginase. However, our previous work provided a new rationale on this treatment design. We showed that vincristine can temporarily disrupt the stromal support to leukemic blasts and retain the L-asparagine depleted microenvironment [8]. However, the high intrinsic asparaginase resistance in T-ALL blasts implies B-ALL chemotherapy regimens may not be as effective as in T-ALL patients [5,9]. Drugs with higher efficacy against T-ALL are urgently needed.

Recent reports showed that another amino acid depleting agent, pegylated arginase I (BCT-100), is effective against T-ALL both *in vitro* and *in vivo*[10,11]. BCT-100 is purified from *B. subtilis* followed by pegylation for prolonging its bio-activity [12]. Arginase breaks down L-arginine into ornithine and urea. This has been proposed as a novel anti-cancer agent because many types of cancer cells cannot synthesize L-arginine [12,13]. However, cells may potentially be resistant to BCT-100 if they express ornithine transcarbamylase (OTC) or they are able to utilize citrulline under an L-arginine starvation setting [14]. As the nutrient-depleting mechanism of BCT-100 is similar to L-asparaginase, we suspect that T-ALL blasts may acquire chemo-resistance to BCT-100 in a manner similar to that of L-asparaginase resistance induced by hMSCs to B-ALL. Therefore we hypothesized that: 1) hMSCs may protect T-ALL blasts from BCT-100 induced cytotoxicity by providing soluble factors involved in L-arginine metabolism; and 2) BCT-100 resistance induced by hMSCs may be overcome by pre-treating MSCs with vincristine.

## Results and discussion

### T-ALL cell lines were BCT-100 sensitive while hMSCs were BCT-100 resistant

**Figure 1:**
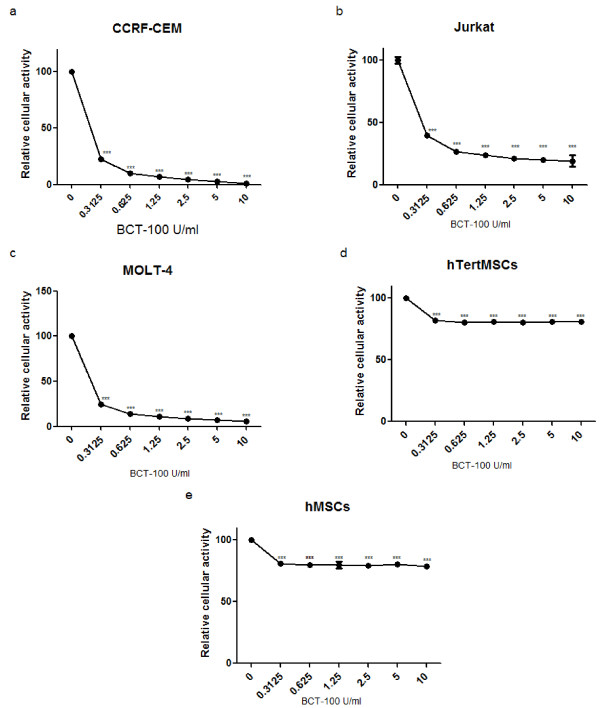
**The cytotoxic assessment of BCT-100 on T-ALL blasts, hTertMSCs and hMSCs showed a dose-dependent response for T-ALL blasts but not MSCs. **(**a**) CCRF-CEM, (**b**) Jurkat, (**c**) MOLT-4, (**d**) hTertMSCs and (**e**) hMSCs were treated with PBS (control) or different doses of BCT-100 (0.15625, 0.3125, 0.625, 1.25, 2.5 and 5 U/ml) for 48 hours, then subjected to XTT analysis. Values of cells viabilities were the representative results derived from 3 consistent experiments with error bars showing standard deviation. ***: *p* < 0.005 compared to control (0 U/ml).

The cell viabilities under BCT-100 treatment were assessed. The tested samples included 3 T-ALL cell lines, CCRF-CEM, Jurkat and MOLT-4; human telomerase reverse transcriptase immortalized MSCs (hTertMSCs); and hMSCs from healthy donors. The dosages of BCT-100 ranged from 0.3125 U/ml to 10 U/ml. All three T-ALL cell lines were sensitive to BCT-100 in a dose-dependent manner. The cell viability of the three T-ALL cell lines dropped below IC_50_ even with the lowest dose of 0.3125U/ml (*p* < 0.005 for all T-ALL cell lines used) (Figure 1a, b and c). On the other hand, the cell viabilities of hTertMSCs and hMSCs remained around 80% even as the BCT-100 dose was stepped up to 10 U/ml (*p* < 0.005) (Figure 1d and e). It is consistent with the previous report that BCT-100 could suppress cell proliferation of both B-ALL and T-ALL [10]. We showed that hMSCs were resistant to BCT-100 and such differential cytotoxicity of BCT-100 to T-ALL blasts and hMSCs might imply a possible symbiosis between T-ALL and hMSCs during arginine starvation stress, similar to the B-ALL blasts/hMSCs symbiosis during asparagine depletion [5].

### hMSCs partly protected T-ALL cells from BCT-100 induced apoptosis

**Figure 2:**
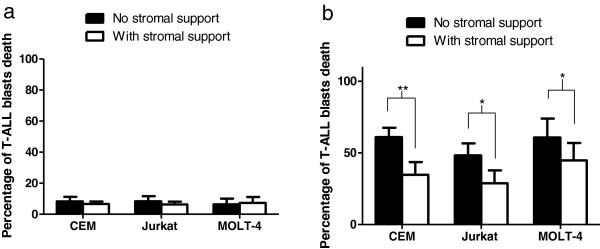
**Apoptosis of T-ALL cell line treated with or without BCT-100 under stromal support/culture alone. **(**a**) CCRF-CEM, Jurkat or MOLT-4 cell lines were cultured for 36 hours with or without hMSCs or hTertMSCs transwell co-culture. Annexin V / FITC Propidium iodide (AV/PI) assay was performed to test the percentage of apoptosis. (**b**) CCRF-CEM, Jurkat or MOLT-4 cell lines were treated with 1 U/ml BCT-100 for 36 hours with or without transwell co-culture with hMSCs or hTertMSCs. AV/PI assay was performed to test the percentage of apoptosis. The average apoptotic percentages of the 3 T-ALL cell lines were significantly increased but such effect was partly attenuated by co-culturing with hTertMSCs and hMSCs from different donors. All experiments were done in triplicate and error bars showed respective standard deviation. *: *p* < 0.05 between T-ALL / hMSCs co-culture and T-ALL culture alone. **: *p* < 0.01 between T-ALL / hMSCs co-culture and T-ALL culture alone.

hMSCs is an important component of bone marrow microenvironment and perivascular niche. It was shown to be resistant to most chemotherapeutic agents [6]. With its intrinsic resistance properties, hMSCs also serve as a supporting niche to cancer cells against chemotherapeutic damage. This protective effect can be found in both solid tumors and leukemia [5,15,16]. In relapsed T-ALL, T-ALL blasts can metastasize to all compartments of the body with grave prognosis. The MSCs residing in the bone marrow microenvironments may provide protection to T-ALL blasts against chemotherapy induced cell death. Such concept is supported by a recent *in vivo* study of BCT-100 in mice [11]. Furthermore, hMSCs can also be found in adipose tissue and around blood vessels as pericytes [17]. Therefore, T-ALL blasts inside the patients’ body may very likely interact with hMSCs not only in the bone marrow microenvironment, but also along the blood vessels. For ensuring efficacy of BCT-100 against T-ALL, it is important to test whether hMSCs and T-ALL cells have symbiotic relationship during BCT-100 treatment. The transwell co-culture system was used to test whether soluble factors in co-culture contributed to protection against BCT-100 in T-ALL blasts. Under ordinary culture conditions, hMSCs did not provide any significant enhancement in survival to T-ALL blasts (Figure 2a). However, hMSCs could protect all 3 T-ALL cell lines used, CCRF-CEM, Jurkat and MOLT-4, against BCT-100 induced apoptosis. Percentage of apoptosis was reduced by as high as 26% in average as shown in CCRF-CEM/hMSCs transwell co-culture, compared to CCRF-CEM alone, *p* < 0.01 (Figure 2b). For Jurkat/hMSCs and MOLT-4/hMSCs transwell co-culture, the average percentages of apoptosis were reduced by about 20%, *p* < 0.05 and 15%, *p* < 0.05 compared to Jurkat and MOLT-4 alone. (Figure 2b) Here we demonstrated that involvement of stroma secreted soluble factors would be sufficient in protecting T-ALL blasts against BCT-100.

We first hypothesized the involvement of cytokines in the hMSCs/T-ALL blasts interaction during BCT-100 treatment. A previous report showed that IL-8 was up-regulated in T-ALL cells refractory to chemotherapy [18]. IL-8 is known to be secreted by both hMSCs and T-ALL cells [19,20]. However, other reports reported that CCRF-CEM, Jurkat, MOLT-4 and primary T-ALL cells do not express IL-8 receptors CXCR1 and CXCR2 [19,21]. It implies that IL-8 works on the cancer micro-environment mainly by enhancing angiogenesis and immune cell migration [19]. Therefore IL-8 may not be an important factor in stromal protection to T-ALL blasts against BCT-100. Therefore, we then investigated whether the possible soluble factors involved are chemicals mainly involve in arginine metabolism.

### Ornithine and citrulline could partly rescue T-ALL cells

**Figure 3:**
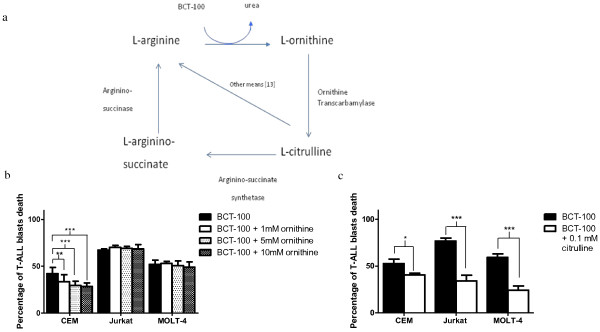
**Apoptosis induced by BCT-100-treated T-ALL cells could be rescue by citrulline but not ornithine*. ***(**a**) The relationship between BCT-100 action and L-arginine metabolism. (**b**) Apoptosis of BCT-100-treated T-ALL cell lines could not be rescued by ornithine in 2 of the 3 T-ALL cell lines tested. CCRF-CEM, Jurkat or MOLT-4 cell lines were treated with BCT-100 with or without ornithine (doses ranged from 1 mM to 10 mM) for 36 hours. Annexin V / FITC Propidium iodide (AV/PI) assay was performed to test the percentage of apoptosis. Only CEM showed a modest reduction of BCT-100 induced apoptosis after the addition of ornithine. The average apoptotic percentages of the 3 T-ALL cell lines were derived from 3 independent experiments for each T-ALL cell line with error bars showing standard deviation. **: *p* < 0.01 between BCT-100 only and BCT-100 + different doses of ornithine. ***: *p* < 0.005 between BCT-100 only and BCT-100 + different doses of ornithine. (**c**) Apoptosis of BCT-100-treated T-ALL cell lines could be rescued by citrulline. CCRF-CEM, Jurkat or MOLT-4 were treated with BCT-100 with or without 0.1 mM citrulline for 36 hours. Annexin V / FITC Propidium iodide (AV/PI) assay was performed to test the percentage of apoptosis. All 3 T-ALL cells showed a reduction in apoptosis after the addition of citrulline. The average apoptotic percentages of the 3 T-ALL cell lines were obtained after triplicated experiment of the corresponding T-ALL cell line with error bars showing standard deviation. *: *p* < 0.05 between BCT-100 only and BCT-100 + 0.1 mM citrulline. ***: *p* < 0.005 between BCT-100 only and BCT-100 + 0.1 mM citrulline.

BCT-100 works by breaking down L-arginine into ornithine and urea. In urea cycle, ornithine can combine with carbamoyl phosphate to form citrulline. Citrulline can then be recycled to L-arginine (Figure 3a). Carbamoyl phosphate and citrulline are both present in blood. Therefore if any cancer cells can utilize ornithine or citrulline during arginine starvation, they may be resistant to the depletion effect of BCT-100 by recycling these intermediate metabolites. In order to determine whether these three T-ALL cell lines can utilize this recycling pathway, the rescuing effect of either ornithine or citrulline on the T-ALL cell lines from BCT-100 induced cell death was investigated. We found that ornithine could reduce the percentage of cell death by about 10% only in CCRF-CEM (*p* < 0.05), but not in Jurkat and MOLT-4 during BCT-100 treatment (Figure 3b). However, the rescue effect for CCRF-CEM was not dose dependent. These results imply that the direct break-down product of L-arginine, ornithine, could not be utilized by T-ALL cells. Although the three T-ALL cell lines had limited or no ability to utilize ornithine to overcome the depletion induced by BCT-100, it is important to determine whether other L-arginine precursors such as citrulline may be able to rescue the blasts. Even citrulline is not generated directly from the catabolic effect of BCT-100, it may be available from other extrinsic sources such as the hMSCs. We found that 0.1mM citrulline could significantly reduce cell death in the CCRF-CEM, Jukrat and MOLT-4 by 12.1%, *p* < 0.01, 42.7%, *p* < 0.005 and 35%, *p* < 0.005, respectively (Figure 3c). The result may contradict a previous report that citrulline could not significantly rescue CCRF-CEM from BCT-100 induced cell death [10]. While in another report, Jurkat cell line could utilize citrulline but not arginino-succinate for growth in the absence of L-arginine *in vitro*[14]. The difference may be related to the dose of citrulline used. The citrulline dose that we used (0.1 mM) was 2.5 fold higher than the average plasma citrulline level in healthy subjects (below 40 μM) [22]. High citrulline level may occur in tissues having high ornithine transcarbamoylase (OTC) activity during BCT-100 treatment. T-ALL cells residing in these sites may acquire resistance to BCT-100 by utilizing citrulline to replenish the depleted arginine. As hMSCs exist not only in bone marrow stroma but also as pericytes around blood vessels, it is important to test whether hMSCs protect T-ALL cells against BCT-100 induced cell death partly by their up-regulation of OTC expression during BCT-100 treatment.

### hMSCs protected T-ALL cell lines against BCT-100 induced cytotoxicity possibly in part by their sustained OTC expression

**Figure 4:**
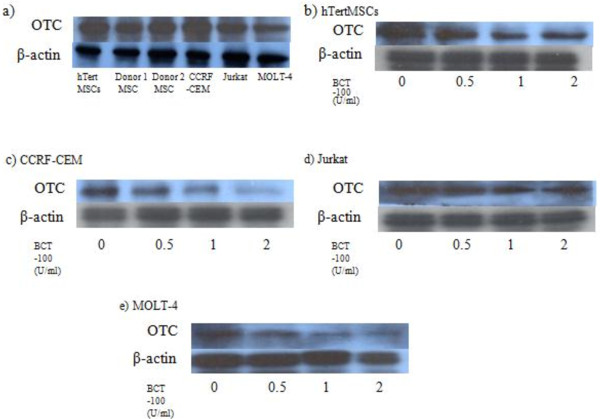
**Ornithine transcarbamylase (OTC) is expressed in both hMSCs and T-ALL cells and BCT-10 treatment could suppress OTC expression significant in T-ALL cells but only modestly on MSCs. **(**a**) Basal expression of OTC in hTertMSCs, hMSCs from 2 healthy donors, CCRF-CEM, Jurkat and MOLT-4. Cells were harvested and respective proteins were extracted from the corresponding cell lines. SDS-PAGE was performed to separate the proteins. OTC and β-actin (internal control) expression were probed by the corresponding antibodies and then visualized by enzyme coupled luminescence (ECL). (**b**) Expression of OTC in hMSCs after BCT-100 treatment. hTertMSCs or hMSCs from a healthy donor were cultured in different doses of BCT-100 (0 U/ml, 0.5 U/ml, 1 U/ml and 2 U/ml) for 36 hours and protein was extracted from the cell culture. SDS-PAGE followed by Western blotting was performed. The expression of OTC and β-actin (internal control) of hTertMSCs is presented in the figure. (**c**), (**d**) and (**e**) Expression of OTC in CCRF-CEM, Jurkat or MOLT-4 after BCT-100 treatment respectively. CCRF-CEM, Jurkat and MOLT-4 were cultured in different doses of BCT-100 (0 U/ml, 0.5 U/ml, 1 U/ml and 2 U/ml) for 36 hours and protein was extracted. SDS-PAGE followed by Western blotting was performed. The expression of OTC and β-actin (internal control) of CCRF-CEM, Jurkat or MOLT-4 is presented in the figures. The most representative results are presented.

Ornithine transcarbamoylase is an enzyme important in urea cycle for arginine synthesis, and it may protect cells against BCT-100 induced arginine depletion. It is known to be highly expressed in liver and gut tissues [12]. However, OTC expression in other tissues has not been documented. In Figure 1, hMSCs were shown to be BCT-100 resistant and the 3 T-ALL cell lines tested were BCT-100 sensitive. It is important to know whether such phenomenon is due to differential expression of OTC in hMSCs and T-ALL cells during BCT-100 treatment. By Western blotting we showed that all hTertMSCs, two hMSCs from healthy donors, CCRF-CEM, Jurkat and MOLT-4 expressed OTC (Figure 4a). This is intriguing as OTC expressing cells are expected to be resistant against L-arginine depletion, which is in contrast to our findings showing the susceptibility of T-ALL cell lines to BCT-100 induced cell death. Furthermore, OTC expressing T-ALL cells lines were expected to have improved survival with ornithine supplement during BCT-100 treatment. Unexpectedly, ornithine failed to rescue Jurkat and MOLT-4 against BCT-100 treatment. Therefore we tested the OTC expression of hMSCs and T-ALL cells during BCT-100 treatment. By Western blotting, we showed that the OTC expression of hMSCs and the 3 T-ALL cells’ decreased significantly after BCT-100 treatment. But such down-regulation occurred in different degrees, the OTC expression in hMSCs but not T-ALL cells remains at a relatively high level (Figure 4b, c, d & e). The suppression of OTC activity among T-ALL cells may hinder the conversion of ornithine to citrulline and eventually leading to the failure in replenishing L-arginine. It is possible that hMSCs protect themselves and also the adjacent T-ALL cells by their sustained OTC expression during BCT-100 treatment by secreting citrulline. Therefore T-ALL cells may utilize extrinsic citrulline to survive despite L-arginine deprivation. In order to disrupt the symbiotic relationship between hMSCs and T-ALL cells during BCT-100 treatment, we tried to test whether we could suppress hMSCs support to T-ALL cells by inhibiting the function of hMSCs with VCR, an anti-microtubule agent that have been shown to suppress the proliferation and function of hMSCs. In addition, vincristine is also a known potent anti-angiogenesis agent that can effectively inhibit blood vessel formation including those in the cancer microenvironment.

### Vincristine pre-treatment on hMSCs could potentially override the protective effect of hMSCs on T-ALL cells during BCT-100 treatment

**Figure 5:**
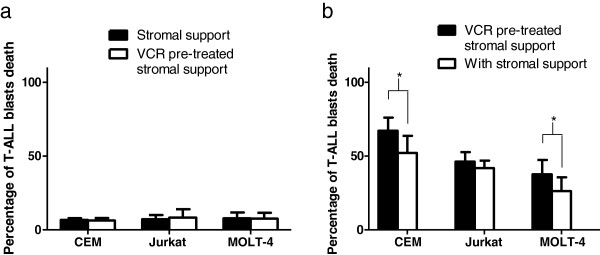
**Apoptosis of T-ALL cell lines treated with BCT-100 under stromal support/ VCR pre-treated stromal support. **The protective effect of hMSCs on T-ALLs during BCT-100 treatment could be abolished by pre-treating hMSCs with vincristine. (**a**) Baseline Apoptosis of T-ALL cell lines was not affected by VCR pre-treatment on hMSCs. CCRF-CEM, Jurkat or MOLT-4 cells were cultured for 36 hours with hMSCs or hTertMSCs transwell co-culture. MSCs were with or without vincristine pre-treatment for 3 days. Annexin V / FITC Propidium iodide (AV/PI) assay was performed to test the percentage of apoptosis. (**b**) VCR pre-treatment on hMSCs could partly re-establish the chemosensitivity of T-ALL cells to BCT-100 as in those without stromal support. hMSCs or hTertMSCs were pre-treated with or without VCR for 3 days, after washing the cells extensively with PBS, CCRF-CEM, Jurkat or MOLT-4 were co-cultured with the pre-treated hMSCs or hTertMSCs. 1 U/ml BCT-100 was used to treat the co-cultures for 36 hours. Then the T-ALL cells were harvested and AV/PI assay was performed to test the percentage of apoptosis. The average apoptotic percentages of the 3 T-ALL cell lines were obtained under transwell co-culture with hMSCs from different donor including hTertMSCs. All experiments were done in triplicate with error bars showing standard deviation. *: *p* < 0.05 between T-ALL / hMSCs co-culture and T-ALL/ VCR pre-treated hMSCs co-culture.

hMSCs are resistant against most of the commonly used chemotherapeutic agents *in vitro*, however, they remain sensitive to drugs that disrupt micro-tubules function such as paclitaxel and VCR [6]. There were also reports showing that intensive combinational chemotherapies cause reduction in number of hMSCs in the form of colony forming unit fibroblasts (CFU-F) isolated from bone marrow aspirates, the dose and synergistic effects of various chemotherapeutic agents may account for the differences [23,24]. Therefore, both anti-microtubules agents and intensive chemotherapy may reduce hMSCs viability, and hence the stromal support to tumor cells in bone marrow micro-environment. Since VCR is a commonly used chemotherapeutic agent and its suppressive effect on MSCs is partly reversible, we selected VCR as our hMSCs suppressor. We hypothesized that by pre-treating hMSCs with VCR, the hMSCs protection to T-ALL blasts against BCT-100 induced cytotoxicity may be suppressed. By AV/PI assay using flow cytometry we showed that pre-treating hMSCs or hTertMSCs with VCR did not increase the percentage of T-ALL blasts apoptosis in all the three T-ALL cell lines (Figure 5a). This excluded the possibility that residual VCR from hMSCs culture medium might cause significant damage to T-ALL blasts. Then we showed that VCR pre-treatment on hMSCs could partly eliminate the protective effect of hMSCs to CCRF-CEM and MOLT-4 cells in co-culture, by 15%, *p* < 0.05 and 11%, *p* < 0.05, respectively, compared to co-culture without vincristine pre-treated hMSCs (Figure 5b). But such maneuver does not benefit Jurkat co-cultured with hMSCs. This may be due to a cell-line-specific phenomenon. The dependence of Jurkat cells to the stromal support during BCT-100 treatment may be low at base line, such that minimal level of arginine or citrulline secreted from the hMSCs may be adequate for rescue. What really account for the difference remains to be investigated further. In summary, the response of the other 2 T-ALLs is in concordance to our previous report that VCR can suppress stromal support to B-ALL blasts against L-asparaginase cytotoxicity [8]. Such approach may help to restore the sensitivity of most T-ALL blasts to BCT-100 cytotoxicity. Our experiment results imply that vincristine may synergize with BCT-100 *in vivo*; this requires further verification by *in vivo* study.

## Conclusions

Differential toxicity of BCT-100 to T-ALL blasts and hMSCs was observed. BCT-100 induced significant cytotoxicity to 3 T-ALL cell lines including CCRF-CEM, Jurkat and MOLT-4 but not hMSCs. With such differential response between T-ALL cells and hMSCs as basis, the interaction of hMSCs and T-ALL blasts during BCT-100 treatment was further investigated. hMSCs could partly rescue all 3 T-ALL cell lines from BCT-100 induced toxicity. While testing for the involvement of L-arginine metabolic pathway substrates in the rescue mechanism, all the 3 T-ALL cell lines tested could utilize citrulline for enhancing survival during BCT-100-induced L-arginine deprivation. On the other hand, only CCRF-CEM could marginally utilize ornithine for survival during BCT-100 treatment. hMSCs and all 3 T-ALL cell lines expressed OTC, which means both hMSCs and T-ALL blasts should be capable of converting ornithine into citrulline and eventually recycling L-arginine even under BCT-100 treatment. However, the expression of OTC could also be suppressed in both hMSCs and T-ALL cell lines during BCT-100 treatment. Despite the decrease in OTC expression, expression in hMSCs remained relatively higher than that of the three T-ALL cell lines. The differential OTC expression between hMSCs and T-ALL cells during BCT-100 treatment suggested that hMSCs could potentially protect T-ALL cells from BCT-100 treatment by their sustained OTC expression. In order to suppress the stromal protection to T-ALL blasts, hMSCs could be pre-treated with vincristine (VCR). The protective effect of hMSCs to 2 of the 3 T-ALL cell lines against BCT-100 were abolished with this approach. This suggests a possible synergy between VCR and BCT-100 in treating T-ALL in the cancer microenvironment, similar to the synergy of VCR and L-asparaginase in ALL chemotherapy protocol. Further investigation using animal studies may help to improve the efficacy of BCT-100, especially when in combination with VCR.

## Material & methods

### Cell culture

Bone marrow mononuclear cells from healthy young adults’ bone marrow transplantation donor were obtained with written informed consent under approval of Institutional Review Board (The Hong Kong University and Hong Kong West Cluster Hospitals). The bone marrow-derived human MSCs were expanded and characterized *in vitro* according to our previous published method [6]. The human telomerase reverse transcriptase immortalized MSCs (hTertMSCs) was a gift from Prof. D. Campana (St Jude Children's Research Hospital, Memphis, Tennessee) [25]. hTertMSCs or hMSCs were cultured in low-glucose Dulbeco's Minimal Essential Medium (DMEM-LG) supplemented with 10% fetal bovine serum (FBS), 1% penicillin/ streptomycin (P/S), and 1% L-glutamine. CCRF-CEM, Jurkat and MOLT-4 are T cell lineage ALL cell lines purchased from American Type Culture Collection (ATCC). The 3 T-ALL cell lines were cultured in RPMI 1640 supplemented with 10% FBS, 1% HEPES, 1% Penicillin / Streptomycin.

### Antibodies and chemicals

BCT-100 was provided by Dr Cheng NM, Paul of Bio-Cancer Treatment Ltd. Vincristine was purchased from Eli Lily (Eli Lily and Company Incorporated, UK). Mouse Anti-human OTC antibody was purchased from Santa Cruz Biotechnology (Santa Cruz, CA). Rabbit anti-human β-actin antibody was purchased from Cell Signaling Technology (Danvers, MA). L-citrulline and L-ornithine were purchased from Sigma-Aldrich Co. (St. Louis, MO, USA).

### Transwell co-culture of MSCs and T-ALL cell lines

hTertMSCs or hMSCs were seeded at the density of 1 × 10^4^ cells/well onto 12-well plates. They were then treated with or without vincristine sulfate (VCR, Eli Lily and Company Incorporated, UK) as previously described [8]. Both MSCs were washed with phosphate buffered saline and then incubated with RPMI 1640 medium supplemented with 10% FBS, 1% HEPES, 1% penicillin/streptomycin and 1% L-glutamine. ALL blasts (1 × 10^5^) (CCRF-CEM, Jurkat or MOL-4) were seeded onto the transwell insert of the 12-well plates seeded with the previously seeded MSCs. MSCs/T-ALL blasts co-culture was treated with or without 1 U/mL BCT-100 for 36 hours.

### Cell viability

Cells were plated at 10^4^ per well in 96 well plate with increasing concentration of BCT-100 (0.3125 U/ml, 0.625 U/ml, 1.25 U/ml, 2.5 U/ml, 5 U/ml and 10 U/ml) for 48 hours at 37°C. Cell viability was determined by a colorimetric method using XTT cell proliferation assay kit according to manufacturer’s instructions (Roche Diagnostics, Switzerland).

### Protein extraction and Western blot analysis

Protein extraction and Western blot analysis were carried out as follow. After treatment, cells were washed with phosphate buffered saline, and then resuspended in lysis buffer (phosphate-buffered saline containing 1% Nonidet P-40, 0.5% sodium deoxycholate and 0.1% SDS) containing the protease inhibitors (100 μg/ml phenylmethylsulfonyl fluoride and 1 mM sodium fluoride). The lysate was incubated on ice for 20 min, and then centrifuged at 13,200 rpm for 20 min at 4°C. Protein concentration was determined using the Bio-Rad protein assay. Whole cell lysate containing 15 μg of protein from each sample were used in immunoblotting, and subsequently the gels were electro-blotted onto PVDF membranes (Immobilon-P, Millipore). Antibodies purchased from Santa Cruz Biotechnology (Santa Cruz, CA) and Cell Signaling Co. (Danvers, MA) were used to detect the proteins of interest. The horseradish peroxidase conjugated antibodies against mouse and rabbit IgG were used as secondary antibodies (Sigma-Aldrich, St. Louis, MO). The secondary antibody binding was detected by ECL Western detection blotting reagents (GE Healthcare, USA) and detected by Fuji Medical X-Ray Film (Fuji, Japan).

### Apoptosis analysis

AV/PI (Annexin V-FITC Kit, Becton, Dickinson and Company, NJ) was used to assess the extent of apoptosis of T-ALL cells after exposure to arginase treatment. T-ALL blasts in transwell co-culture were harvested, washed and then re-suspended in 1X AV binding buffer provided by the commercial kit. The cells were labeled with AV-FITC and propidium iodide (PI) following the manufacturer's instruction and analyzed by flow cytometry. Flow cytometric analysis was performed with a BD LSR II (BD Biosciences, San Jose, CA). Ten thousand events per sample were collected into list mode files and analyzed by FlowJo.

### Statistical analysis

Experiments were performed for 3 times with consistent results. Comparisons between mean values were made using two way's analysis of variance (ANOVA) or paired Student t test (one-tailed). The differences were considered statistically significant when *p* < 0.05.

## Abbreviations

T-ALL: T-lineage acute lymphoblastic leukemia; BCT-100 (brand name): Pegylated arginase; hMSCs: Human mesenchymal stromal cells; XTT assay: 2,3-bis-(2-methoxy-4-nitro- 5-sulfophenyl)-2H-tetrazolium- 5-carboxanilide assay, vincristine (VCR); AV/PI assay: Annexin V / propidium iodide assay; OTC: Ornithine transcarbamylase; hTertMSCs: Human telomerase reverse transcriptase immortalized mesenchymal stromal cells.

## Competing interests

The authors declare no competing interests.

## Authors’ contributions

FKL and CGC designed the experiments, wrote and revised the manuscript. FKL performed the experiments. Both authors read and approved the final manuscript.
